# Supplementary material for: Asynchronous magnetic resonance elastography: Shear wave speed reconstruction using noise correlation of incoherent waves
Source: Magn Reson Med. 2022 Oct 27;89(3):990–1001. doi: 10.1002/mrm.29502 (PMC9792433; doi:10.1002/mrm.29502)
Supplement: Supplementary file 1 — DATA S1 MATLAB code used to generate the numerical simulations of Figure 1 [file MRM-89-990-s002.zip › k-Wave/helpfiles/revolve2D.html]

revolve2D :: Functions (k-Wave)


# revolve2D

Form 3D matrix from revolution of 2D matrix.

## Syntax

```
mat3D = revolve2D(mat2D)
```

## Description

`revolve2D` revolves the values of a 2D matrix about the x axis (or matrix rows) to form a 3D matrix. A single point is taken as the x-axis origin, thus for an input matrix of size m by n, the output matrix will be m by (2n-1) by (2n-1). Values outside the interpolation range are set to zero. An example of revolving a 2D triangle is shown below.

```
% create a triangle in a 2D grid
triangle = tril(ones(10, 10));

% rotate in 3D
triangle_3D = revolve2D(triangle);

% plot the revolved triangle
voxelPlot(triangle_3D);
camup([0, -1, 0]);
```


## Inputs

|  |  |
| --- | --- |
| `mat2D` | 2D input matrix |

## Outputs

|  |  |
| --- | --- |
| `mat3D` | 3D output matrix |

## See Also

`interp1`
